# Optimization of diazinon biodegradation from aqueous solutions by *Saccharomyces cerevisiae* using response surface methodology

**DOI:** 10.1186/s13568-017-0366-5

**Published:** 2017-03-21

**Authors:** Mohammad H. Ehrampoush, Abbas Sadeghi, Mohammad T. Ghaneian, Ziaeddin Bonyadi

**Affiliations:** 1 Department of Environmental Health Engineering, Environmental Science and Technology Research Center, Shahid Sadoughi University of Medical Science, Yazd, Iran; 2 Department of Environmental Health, School of Health, Mashhad University of Medical Sciences, Mashhad, Iran

**Keywords:** *Saccharomyces cerevisiae*, Diazinon, Biodegradation, RSM

## Abstract

Diazinon is an organophosphate compound that inhibits the activity of acetylcholinesterase. Standards of the World Health Organization and Environmental Protection Agency for diazinon concentration in water are 0.1 and 9 × 10^−6^ mg/L, respectively. The aim of this study was the optimization of diazinon biodegradation from aqueous solutions by *Saccharomyces cerevisiae* using the response surface methodology (RSM). Harvested cells of *S. cerevisiae* were locally purchased from the Iranian Research Organization for Science and Technology. To obtain the optimum condition for diazinon biodegradation using RSM, input parameters included the initial concentration of diazinon (0.01–10 mg/L), concentration of *S. cerevisiae* (0.5–5%), pH (4–10), and retention time (1–30 h). The research study had a central composite design where one of the methods was RSM. According to the results, the observed values of the removal efficiency of diazinon were variable in the range of 23–96. The highest removal rate was obtained as 96% under the initial diazinon concentration of 2.5 mg/L, *S. cerevisiae* concentration of 3.88%, pH of 5.5, and retention time of 22.75 h. The results displayed that the removal efficiency of diazinon had a direct relationship with the concentration of *S. cerevisiae* and retention time, and an inverse relationship with pH and the initial concentration of diazinon. We can conclude that *S. cerevisiae* has the ability to remove diazinon with the lowest cost and a high efficiency.

## Introduction

The pollution of water resources by pesticides is considered an environmental concern. The use of pesticides in the agricultural sector has increased due to the population growth and increasing demand for agricultural products and foodstuffs (Khazaei et al. 2010; Arjmandi et al. 2010). Several applications of pesticides in agriculture can cause environmental issues such as contamination of water, soil, and the food. Pesticides include organophosphate, carbamate, and pyrethroid, and the organophosphorus compounds are the largest and most diverse pesticides. Because of their effect on a wide range of pests and also their low cost, organophosphate pesticides are used by farmers more than other pesticides (Shayeghi et al. 2008). Different types of organophosphorus compounds inhibit the cholinesterase enzyme and therefore cause a range of toxic effects. These effects include headaches, vomiting, and respiratory tract problems. Depending on the chemical nature of organophosphorus compounds, inhibiting the cholinesterase may be reversible or irreversible (Hashemian et al. 2015). Diazinon is an organophosphate compound with the chemical formula of C_12_H_21_N_2_O_3_PS (Hunter et al. 1985; WHO 1998). According to the standard of the European Union, the maximum allowable concentration for the total pesticide residue and for each pesticide in drinking water sources is 0.5 μg/L and 0.0001 mg/L, respectively (Cai and Yun 2008). World Health Organization (WHO) and the Environmental Protection Agency (EPA) standards for the value of diazinon in drinking water are 0.1 μg/L and 9 × 10^−6^ mg/L, respectively (Goodrich et al. 1989). The maximum allowable concentrations of diazinon in drinking water in Australia, Canada, Germany, England and America are 20, 10, 0, and 0.6 μg/L, respectively (Readman et al. 1992). Different methods such as filtration, ozonation, and adsorption using the granular activated carbon are used for the removal of pesticides from aqueous solutions, but these methods have been associated with problems such as high capital and operating costs, saturation of activated carbon, and production of toxic substances (Reynolds et al. 1989; Lai et al. 1995; Jiang and Adams 2006; Ormad et al. 2008). *Saccharomyces cerevisiae* is a single-cell and non-pathogenic yeast (Cabral et al. 2003; Teixeira et al. 2007). It is considered for the biological removal of diazinon due to advantages such as safety, low cost, simplicity, widespread distribution, rapid growth of cells, and easy cultivation. The main advantage of *Saccharomyces cerevisiae* is its selectivity in active transport and competitiveness in biological absorption (Goksungur et al. 2005; Peinado and Morenoa 2006; Yarke-Salkhori et al. 2011). Mutagenicity studies indicated that diazinon does not have negative effects on the metabolic system of *S. cerevisiae* (Mohn 1973; Fahrig 1974), and only at concentrations greater than 50,000 ppm can it have negligible toxic effects on *S. cerevisiae* (Bianchi et al. 1994). Sadeghi et al. (2014) used *S. cerevisiae* for the biological removal of carmoisine and Reactive Black 5 dyes (Sadeghi et al. 2014). Fabrizio et al. (1983) demonstrated that this type of yeast can biologically remove the folpet and metalaxyl fungicides and the pyrethroid and deltametrin insecticides (Fabrizio et al. 1983). Also, Tijana and Rada (2015) reduced the chlorpyrifos-methyl pesticide with this method (Tijana and Rada 2015), and Cabras and Angioni (2000) and George et al. (2014) carried out a similar study (Cabras and Angioni 2000; George et al. 2014). The technique of experimental design is a useful tool that uses statistical models to optimize the interaction between different parameters. Using the response surface methodology (RSM), we can study the interaction between two or more parameters (Yi et al. 2012). The present study was conducted with the aim of optimizing diazinon biodegradation from aqueous solutions by *Saccharomyces cerevisiae* using RSM.

## Materials and methods

### Chemicals

All chemicals used in the experiments were reagent grade. All solutions were prepared with distilled water. Diazinon was obtained from Sigma-Aldrich.

### Microorganism

The harvested cells of *S. cerevisiae* [Persian-type culture collection (PTCC):5052] were locally purchased from the Iranian Research Organization for Science and Technology, Tehran, Iran. In this experiment, yeast was prepared at the concentration of 5%. For this purpose, 5 g of yeast was suspended in 100 mL of toxic substance solution.

### Preparation of reaction mixtures

**Fig. 1:**
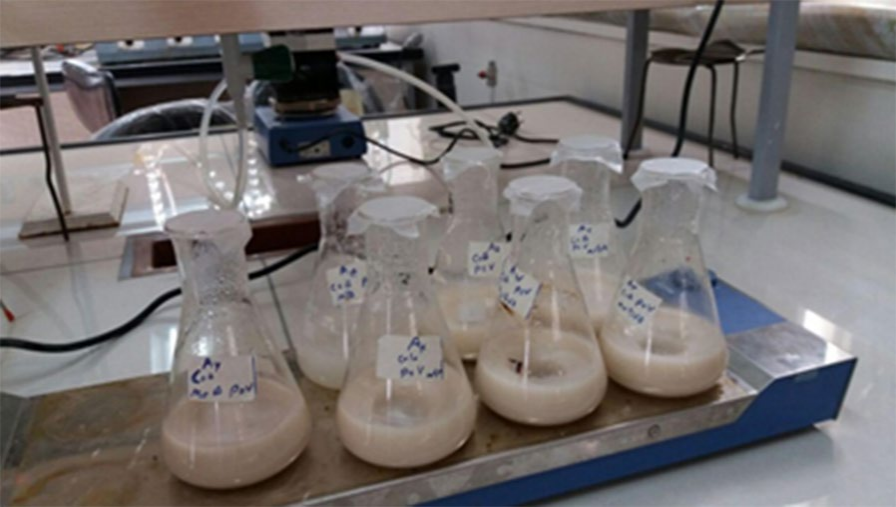
Biodegradation of diazinon using wet cells of *S. cerevisiae* at different pH, concentration and reaction times

In this study, 100 cc of reaction mixture at the concentration of 0.01–10 mg/L of diazinon and 0.5–5% of *S. cerevisiae*, with the pH of 4–10 and retention times of 1–30 h were prepared. The experiments were performed at the fixed agitation speed of 120 rpm and room temperature (28 ± 2 °C). Figure 1 shows the reaction mixture with the volume of 100 mL at different pH concentration and reaction times.

### Analytical methods

Ten microliter of sample was taken from each Erlenmeyer flask at definite time intervals. Samples were centrifuged at 4000 rpm for 10 min to remove the medium. The diazinon concentration was measured using isocratic reverse-phase HPLC (model of KNAUER, Germany) and the EZ-chrome software with a UV–Vis absorbance detector at the wavelength of 269 nm in column C-18. The operation was conducted at room temperature with a mobile phase containing methanol: water (50:50) with the column size 4.6 × 150 mm, the flow rate of 0.8 mL/min, and the maximum pressure of 40 MPa (APhA et al. 1998).

### Experimental design and statistical analysis

The experiment was designed based on RSM. As shown in Table 1, four parameters were selected for this study, including the initial concentration of diazinon (mg/L), reaction time (h), concentration of *S. cerevisiae* (%), and pH which was high, low, and medium for the three levels coded +1, 0, and −1, respectively. Moreover, assisting points coded +α and −α were considered for model validation based on the models.

**Table 1:** Experimental ranges and levels of independent parameters according to RSM design

Parameters	Symbol	Levels				
		−α	−1	0	+1	+α
Concentrations of diazinon (mg/L)	A	0.01	2.5	5	7.5	10
Reaction time (h)	B	1	8.25	15.5	22.75	30
Concentration of *S. cerevisiae* (%)	C	0.5	1.63	2.75	3.88	5
pH	D	4	5.5	7	8.5	10

In this study, 30 runs were performed using RSM with 16 real points, 8 pivot points, and 6 central focal point. The quadratic model for the variables is presented below:

1 $${\text{Y}} =\upbeta_{0} + \sum\limits_{i = 1}^{k} {\upbeta_{i} x_{i} } + \sum\limits_{i = 1}^{k} {\upbeta_{ii} x_{i}^{2} } + \sum\limits_{{1{ \le }i{ \le }j}}^{k} {\upbeta_{ij} x_{i} x_{j} }$$

where Y, β_0_, β_i_, β_ii_, β_ij_ and x_i_ or x_j_ are the predicted response, the constant coefficient, regression coefficients for linear effects, quadratic coefficients, interaction coefficients, and the coded values of the parameters, respectively. The fit of the models was evaluated by determining the coefficients (R^2^) and adjusted R^2^ (R^2^adj) (Soumasree et al. 2012).

## Results

In this study, the effect of parameters such as concentration of diazinon, concentration of *S. cerevisiae*, reaction time, and pH were studied on the removal efficiency of diazinon. The summary of the results of the study are presented in Table 2.

**Table 2:** Experimental design and response values at different runs of diazinon removal

Run order	Parameters				The removal efficiency of diazinon (%)
	A	B	C	D	
1	5	15.5	5	7	58.34
2	10	15.5	2.75	7	27
3	2.5	22.75	1.63	8.5	47
4	5	15.5	0.5	7	34
5	2.5	8.25	1.63	5.5	5
6	7.5	22.75	1.63	5.5	45
7	5	15.5	2.75	4	60
8	7.5	22.75	1.63	8.5	32
9	5	15.5	2.75	10	27
10	5	15.5	2.75	7	43
11	5	15.5	2.75	7	44
12	2.5	22.75	3.88	8.5	65
13	5	15.5	2.75	7	27
14	7.5	22.75	3.88	5.5	48
15	2.5	8.25	3.88	8.5	44
16	5	15.5	2.75	7	38.02
17	5	15.5	2.75	7	43.86
18	5	30	2.75	7	85
19	2.5	22.75	3.88	5.5	96
20	2.5	8.25	3.88	5.5	58.93
21	7.5	8.25	3.88	5.5	33
22	7.5	8.25	1.63	5.5	26
23	5	15.5	2.75	7	35
24	7.5	22.75	3.88	8.5	40
25	2.5	8.25	1.63	8.5	32
26	7.5	8.25	3.88	8.5	30
27	5	1	2.75	7	25
28	7.5	8.25	1.63	8.5	23
29	0.01	15.5	2.75	7	87
30	2.5	22.75	1.63	5.5	65

**Fig. 2:**
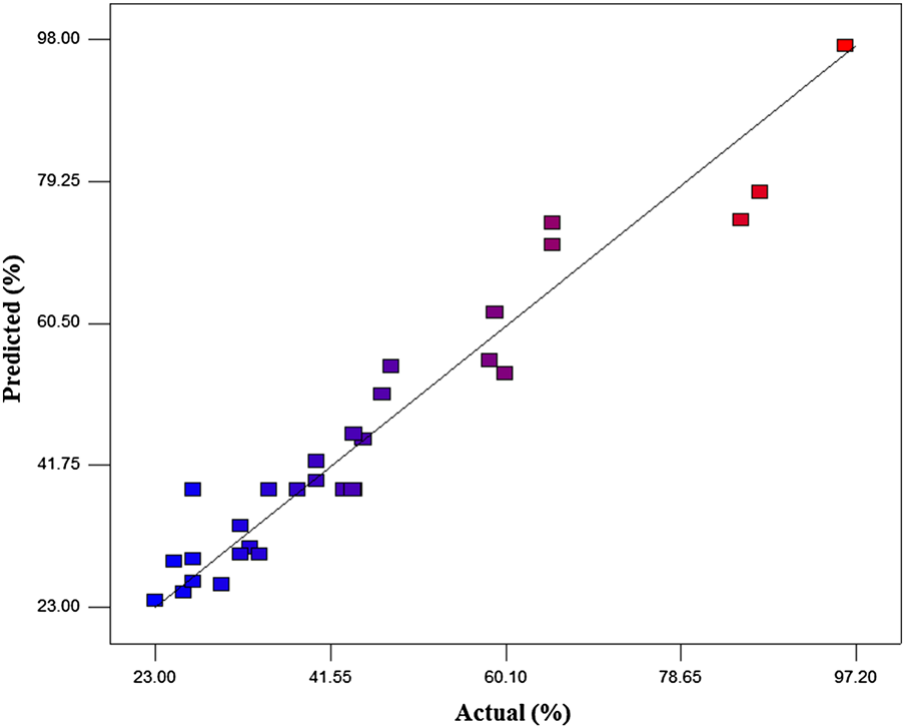
Actual and predicted removal of diazinon

Table 3 shows the regression results of quadratic model for the removal efficiency of diazinon; Fig. 2 demonstrates the correlations between the actual and predicted removal of diazinon.

**Table 3:** ANOVA of the quadratic model for the removal efficiency of diazinon

Source	Sum of squares	Degree of freedom	Mean square	F-value	P value
Model	9889.93	14	706.42	14.45	<0.0001
Residual	733.30	15	48.89		
Lack of fit	509.34	10	50.93	1.14	0.4715
Pure error	223.96	5	44.79		
Total	10623.23	29			
R^2^ = 9310 Adj R^2^ = 0.8665 Pred R^2^ = 0.6935 Adeq precision = 14.84

The quadratic model is shown in Eq. 2 in terms of coded factors of the removal efficiency of diazinon (*Y*):

2 $$\begin{aligned} {\text{Y}} & = + 38.48-12 {\text{A}} + 11.29 {\text{B}} + 6.4 {\text{C}} - 6.87 {\text{D}} - 2.82 {\text{AB}} - 3.43 {\text{AC}} + 2.81 {\text{AD}} + 0.94 {\text{BC}} - 2.57 {\text{BD}} \\ & \quad - 0.93 {\text{CD}} + 3.78 {\text{A}}^{ 2} + 3.28 {\text{B}}^{ 2} + 1.07 {\text{C}}^{2} + 0.4 {\text{D}}^{2} \\ \end{aligned}$$

**Fig. 3:**
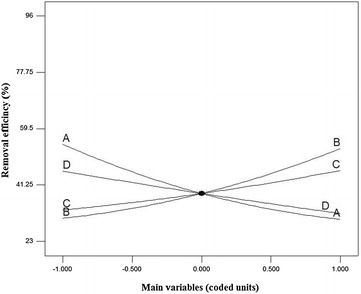
Response surface (perturbation) showing the effect of the main variables (*A*, *B*, *C* and *D*) on the removal efficiency of diazinon

As can be seen in the equation, each model has a fixed part and a variable part. On the basis of Eq. (2), the removal efficiency of has have been 38.48% that is affected by different factors. The main effects of A, B, C, and D have the coefficients of −12.12, +11.29, +6.4, and −6.87, respectively. The main effect belongs to the variable which is shown with the coefficient of +11.29. The highest interaction effect belongs to the AC with the coefficient of 3.43, and the highest square effects of the factors belong to the A^2^ with the coefficient of +3.78. The effects of the main variables (between −1 and +1 levels) on the removal efficiency of diazinon are demonstrated in Fig. 3. With regard to Fig. 3, it is necessary to note that to describe the effect of one factor on a response, other variables are fixed at the zero level. For example, when the variable of reaction time or B increases from level −1 (1 h) to +1 (30 h), the other three variables of the concentrations of diazinon or A (5 mg/L), concentration of *S. cerevisiae* or C (2.75%), and pH or D (7) are at the zero level. The optimized values for pH, concentrations of diazinon, concentration of *S. cerevisiae* and reaction time were found to be 5.51, 2.52 mg/L, 3.84% and 22.6 h, respectively. At these conditions, the predicated diazinon removal percentage was more than 96.1% with desirability 1. Conduction of similar experiments at specified optimum conditions reveal the high repeat ability of method for prediction of real removal percentage with relative deviation less than 2%.

## Discussion

According to the results presented in Table 2, the maximum and minimum removal efficiency of diazinon were 96 and 23%, respectively. Results showed that the observed values of the removal efficiency of diazinon were in the range of 23–96 which was not different from the values predicted by the model (RSM). The findings of this study (using Eq. 2) showed that this treatment process was able to remove 96% of diazinon from aquatic environments. This process underwent the main, interaction, and square effects. Each of these effects have coefficients with positive or negative signs that indicate positive or negative effects are on removal efficiency. The best model to fit the experimental data with independent variables was the quadratic model. ANOVA was used to determine the significance of the model (P values < 0.05). Overall, results showed that this process was significance (P values < 0.0001). Results presented in Table 3 showed that R^2^, justified R^2^, and adequacy precision were 0.931, 0.8665, and 14.842, respectively. Figure 3 indicated that with increasing the parameters from −1 level (2.5 mg/L) to +1 level (7.5 mg/L), the removal efficiency of diazinon reduced significantly. There is an inverse relationship between the removal efficiency of diazinon and its initial concentration, so that with increasing the diazinon concentration from −1 level to +1 level, its removal efficiency decreased 24.25% while the reaction time, pH, and concentration of yeast were at the zero level (*P* value < 0.0001). This is because with increasing the concentration of diazinon, adsorption sites on yeast walls are saturated and decrease the absorption capacity (Mahmoud 2016). Mahmoud (2016) confirmed that with the use of *S. cerevisiae*, the removal efficiency of reactive dye decreased with increasing the concentration of its pollutant (Mahmoud 2016). Studies show that numerous bacteria are involved in the degradation of diazinon (Kristina et al. 2008). The hydrolase enzyme in bacteria plays a major role in the biodegradation of diazinon. It can biodegrade alkyl and aryl bonds of P-O in organophosphates (Gunner and Zuckerman 1968; Kristina et al. 2008). Abasalt et al. (2014) showed that *Pseudomonas plecoglossicida* can use diazinon as a carbon and phosphorus source and biodegrade it. Mahiudddin et al. (2014) concluded that *Pseudomonas peli*, *Burkholderia caryophylli* and *Brevundimonas diminuta* can completely biodegrade diazinon in the concentration of 20 mg/L (Mahiudddin et al. 2014). According to Fig. 3, there is a direct relationship between the removal efficiency of diazinon and reaction time, so that with increasing the reaction time from −1 level to +1 level, removal efficiency increased 43.39% while the initial concentrations of diazinon, pH, and the concentration of yeast were at the zero level (P value < 0.0001). This positive effect (Eq. 2) has been shown by the coefficient of +11.29 for parameter B. Mariusz et al. (2009) indicated that Serratia and Pseudomonas can successfully biodegrade diazinon (at the concentration of 50 mg/L for the period of 42 days) by 83 and 87%, respectively (Mariusz 2009). Sadeghi et al. (2014) showed that *S. cerevisiae* can biodegrade Carmoisine and Reactive Black 5 Dyes within 24 h. Therefore, the maximum removal of carmoisine and Reactive Black 5 Dyes was 85 and 53%, respectively (Sadeghi et al. 2014). Bumpus et al. (1993) reported the ability of *Phanerochaete chrysosporium* to degrade 27.5% of chlorpyrifos during the 18-day incubation (Bumpus et al. 1993). The results demonstrated a direct relationship between the removal efficiency of diazinon and the concentration of *S. cerevisiae*, so that the removal efficiency of diazinon (while the initial concentration of diazinon, pH, and reaction time were at the zero level) increased by 27.8% over time from −1 level to +1 level (P value < 0.05). Zaharia et al. (2013) indicated that a 5% concentration of *S. cerevisiae* can significantly biodegrade the DINOCAP and DNOC pesticides. The findings of this study (Fig. 3) suggest that there is an inverse relationship between the removal efficiency of diazinon and pH. Accordingly, with increasing the pH from −1 level to +1 level, the removal efficiency of diazinon decreases by 30% while the reaction time, initial concentration of diazinon, and the concentration of *S. cerevisiae* were at the zero level (P value < 0.0001). pH is one of the important parameters influencing chemical reactions and biological aqueous solutions. It affects the surface charge of absorbents and the ionization degree of absorbate (Sadeghi et al. 2015). *S. cerevisiae* has carboxyl, phosphonate, and amine groups which include negative, negative, and positive charges, respectively. Amine groups are mainly found in proteins and bio-mass and are also more active than other groups in biosorption (Sadeghi et al. 2015). At a low pH, active sites (amino groups) of *S. cerevisiae* are protonated and the density of positive charge in the absorbent surface increases (Sadeghi et al. 2015). If the adsorption phenomenon is involved alone, a high adsorption must be observed in an alkaline pH, while a high adsorption must observed in an acidic pH (Konstantinos et al. 2012; Sadeghi et al. 2015). Kristina et al. (2008) showed that the removal efficiency of diazinon increases by microorganisms at a low pH (Kristina et al. 2008). Aksu (2003) demonstrated that the efficiency of *S. cerevisiae* in reactive dye removal increased significantly at a low pH.

No similar study has been published on the use of *S. cerevisiae* in the removal of diazinon. Results showed that the removal efficiency of diazinon by *S. cerevisiae* highly depends on pH. The maximum removal rates have been observed at an acidic pH. The removal efficiency of diazinon has a direct relationship with the concentration of *S. cerevisiae* and reaction time, and an inverse relationship with pH and initial concentration of diazinon. We can conclude that *S. cerevisiae* has the ability to remove diazinon with the lowest cost and a high efficiency.

## Abbreviations

RSM: response surface methodology
WHO: World Health Organization
EPA: Environmental Protection Agency
PTCC: Persian-type culture collection
